# General requirements for stem cells

**DOI:** 10.1111/cpr.12926

**Published:** 2020-11-04

**Authors:** Jie Hao, Aijin Ma, Lei Wang, Jiani Cao, Si Chen, Liu Wang, Boqiang Fu, Jiaxi Zhou, Xuetao Pei, Yu Zhang, Peng Xiang, Shijun Hu, Qiyuan Li, Yong Zhang, Yingjie Xia, Huanxin Zhu, Glyn Stacey, Qi Zhou, Tongbiao Zhao

**Affiliations:** ^1^ State Key Laboratory of Stem Cell and Reproductive Biology National Stem Cell Resource Center, Institute for Stem Cell and Regeneration, Institute of Zoology, Chinese Academy of Sciences Beijing China; ^2^ Chinese Society for Stem Cell Research Shanghai China; ^3^ China National Institute of Standardization Beijing China; ^4^ Beijing Technology and Business University Beijing China; ^5^ China National Institute of Metrology Beijing China

**Keywords:** detection control, general requirements, quality control, stem cells, waste disposal

## Abstract

The standard ‘*General requirements for stem cells*’ is the first set of general guidelines for stem cell research and production in China, jointly drafted and agreed upon by experts from the Chinese Society for Stem Cell Research. This standard specifies the classification, ethical requirements, quality requirements, quality control requirements, detection control requirements and waste disposal requirements of stem cells, which is applicable to stem cell research and production. It was firstly released by the Chinese Society for Cell Biology on 1 August 2017 and was further revised on 30 April 2020. We hope that publication of these guidelines will promote institutional establishment, acceptance, and execution of proper protocols, and accelerate the international standardization of stem cells for clinical development and therapeutic applications.

## 1 SCOPE

This document specifies the classification, ethical requirements, quality requirements, quality control requirements, detection control requirements and waste disposal requirements of stem cells.

This document is applicable to stem cell research and production.

## 2 TERMS AND DEFINITIONS

The following terms and definitions apply to this document.

### 2.1 Ethical committee

A specialized organization responsible for assessing and reviewing ethical issues involved in scientific research.

### 2.2 Informed consent

The process by which an individual or its designated legal representative voluntarily confirms willingness to donate biological material for research‐related purposes, after having been informed of all aspects of the potential research that are relevant for the decision to donate.

[SOURCE: ISO 21709:2020, 3.11]

### 2.3 Stem cell

Cells with the capacity for self‐renewal, and which can differentiate into one or more different types of specialized functional cells.

### 2.4 Totipotent stem cell

Stem cells that can differentiate to form a complete and intact new organism.

### 2.5 Pluripotent stem cell

Stem cells that can self‐renew indefinitely in vitro and possess the potential to differentiate into cells of the three embryonic germ layers, namely the endoderm, mesoderm and ectoderm.

Note: Pluripotent stem cells include but are not limited to embryonic stem cells, somatic cell nuclear transfer (SCNT)‐derived embryonic stem cells, and induced pluripotent stem cells, etc.

### 2.6 Embryonic stem cell

The primary undifferentiated cells derived from the inner cell mass of a blastocyst or an early‐stage pre‐implantation embryo that can self‐renew indefinitely in vitro and possess the potential to differentiate into cells of the three embryonic germ layers.

### 2.7 Somatic cell nuclear transfer (SCNT)‐derived embryonic stem cell

Embryonic stem cells derived from the inner cell mass of a blastocyst or an embryo produced by the in vitro transfer of a donor somatic cell nucleus into an enucleated oocyte.

### 2.8 Induced pluripotent stem cell

Pluripotent stem cells similar to embryonic stem cells in properties but which are derived from somatic cells through artificial reprogramming by introducing genes or proteins, or via chemical or drug treatments.

### 2.9 Adult stem cell

Undifferentiated stem cells located within different adult tissues.

### 2.10 Harvest

The process of obtaining biological samples such as tissues and/or cells from donors.

### 2.11 Separation

The process of obtaining target cells from biological samples.

### 2.12 Cryopreservation

The process by which cells are maintained in an inactive state at a low temperature (<−196°C) so they can be revived later.

### 2.13 Resuscitation

The process whereby cryopreserved cells return to their normal growth state.

### 2.14 Expansion

The process of increasing the numbers of cells upon culture.

### 2.15 Differentiation

The process of gradually converting stem cells into a defined cell state/fate with different morphology and functional characteristics.

### 2.16 Stem cell bank

A legal entity or part of a legal entity that performs biobanking of different source of stem cells and their associated information.

[SOURCE: ISO 20387:2018, 3.5]

### 2.17 Cell purity

The percentage of a particular cell type with defined specific biological characteristics, such as cell surface markers, genetic polymorphisms and biological activities, within a cell population.

### 2.18 Cell viability

The percentage of cells that are alive and metabolically normal within a cell population.

Note: Cell viability can vary over time in culture and may be measured by metabolic activities (eg, esterase activity, Thiazole blue method based on the determination of succinic dehydrogenase [MTT]), apoptosis markers, cellular redox potential, membrane potential, proliferation rate (eg, DNA content), mitochondrial function and membrane integrity, etc.

### 2.19 Stem cell self‐renewal

The ability of stem cells to divide symmetrically, forming two identical daughter undifferentiated stem cells; or divide asymmetrically, forming one daughter cell which can proceed irreversibly to a differentiated cell lineage and ultimately lead to specialized functional differentiated cells, while the other daughter cell still retains the undifferentiated characteristics of the parental stem cell.

### 2.20 Stem cell differentiation potential

The ability that stem cells can produce other types of cells with stably different morphologies, structures and biological functions after cell division.

## 3 CLASSIFICATION

### 3.1 According to the sources of stem cells

Stem cells can be divided into embryonic stem cells and adult stem cells.

### 3.2 According to the potential of differentiation

Stem cells can be divided into totipotent stem cells, pluripotent stem cells, multipotent stem cells, oligopotent stem cells and unipotent stem cells.

## 4 ETHICAL REQUIREMENTS

1. Any organization that performs non‐profit research and/or for‐profit production of stem cells shall establish an ethics committee responsible for the oversight and evaluation of related ethical issues, including primary review, follow‐up review and inspection review mechanisms.
2. The ethical standards for stem cell‐related operations shall be established in accordance with the legal and regulatory requirements of the state.

## 5 QUALITY REQUIREMENTS

### 5.1 General requirements

Appropriate quality control criteria and corresponding methods for establishing stem cell qualities, including biological characteristics, safety, stability and efficacy shall be established and strictly implemented, according to the specific types of stem cells.

### 5.2 Biological characteristics

Biological characteristics criteria of stem cells shall be established with, including but not limited to gene expression biomarkers, self‐renewal capacity, differentiation potential, cellular morphology, genetics and metabolic isoenzyme activities.

### 5.3 Safety

Safety criteria of stem cells shall be established with, including but not limited to microbial tests (bacterial, fungal), mycoplasma tests, tests for intra‐ and extra‐cellular pathogenic factors, endotoxin tests, immunological response tests, tumorigenicity tests, tests for residual quantities of culture medium and xenogeneic ingredients.

### 5.4 Stability

Stability criteria of stem cells shall be established with, including but not limited to the culture density/concentration, purity, viability and biological activities of the stem cells.

### 5.5 Efficacy

Efficacy criteria of stem cells shall be established with, including but not limited to the expression of specific genes and proteins, the secretion of specific cellular factors, the differentiation potential, as well as the structure and physiological function of the stem cells and their differentiated cells.

## 6 QUALITY CONTROL

### 6.1 General requirements

1. Stem cell operators shall possess appropriate professional knowledge and corresponding skills, and undergo regular trainings and assessments to stay updated on the latest developments.
2. Equipment shall meet the requirements of stem cell research and production as well as meet the requirements of metrological criteria.
3. All raw materials, auxiliary materials and waste disposal shall be monitored and recorded.
4. According to the unique characteristics of the stem cells, the standard operating procedures and quality control criteria for the harvesting, isolation, expansion, storage, transportation and testing, etc shall be established and regularly reviewed and revised.
5. The operating environment and conditions, including but not limited to temperature, humidity and cleanliness, as required by each unique stem cell type, shall be monitored and regulated.
6. All relevant documents shall be authentic, complete, traceable and maintained.

### 6.2 Production

#### 6.2.1 Requirements for raw materials

1. The sources, harvesting and isolation of stem cells shall comply with the domestic laws and ethical regulations.
2. The harvesting of human gametes and embryos for stem cell research shall be strictly inspected and reviewed.
3. Written and valid informed consent shall be signed by the donor. The content of informed consent shall include, but is not limited to, the research purpose, potential research and clinical applications under appropriate conditions, feedback on unexpected discoveries, potential commercial value and other issues affiliated to the research. Mechanisms for protecting the personal data and privacy of the donor shall be established.
4. Organizations performed stem cell research and/or production shall establish and implement the standards for donor evaluation. Each stem cell line shall be accompanied with detailed documentation on the harvest methods and donor information, including but not limited to medical history and family history of any medical conditions. The blood type (eg, ABO, Rh) and human leucocyte antigen (HLA) alleles of the donor shall be documented if necessary.
5. The origin of stem cells shall be traceable by referring to the relevant informed consent and/or their genome and functional data.
6. Auxiliary materials such as culture medium and growth factors shall meet the corresponding quality control requirements. The auxiliary materials can be inspected and laboratory tested if necessary.
7. When using animal serum, they shall be free of contamination by viruses of animal origin. Serum from animals in geographical regions with prion epidemics (eg, bovine spongiform encephalopathy) shall be prohibited.
8. If human blood components are used in the culture medium, including but not limited to albumin, transferrin and various cytokines, the source, batch number and quality verification reports shall be provided. State‐approved products shall be used as much as possible.

#### 6.2.2 Preparation

##### Harvesting and separation

1. Documentation for the unique identification of every stem cell sample shall be established.
2. Precautionary measures shall be taken to protect the health and safety of both donor and operator during the harvesting and separation procedures.

##### Expansion

1. During the process of cell expansion, cross‐contamination and mislabelling of cell lines shall be avoided, and risk mitigation measures shall be established.
2. During stem cell expansion, the name of each cell line, passage number, culture conditions, date and name of operator shall be clearly recorded.

##### Differentiation

1. During stem cell differentiation, the starter batch of cells, equipment, culture conditions and operation procedures shall be defined and documented. Differentiation of stem cells shall be reproducible with the same differentiation protocol.
2. The differentiated cells from stem cells shall be clearly defined using characteristics including but not limited to morphology, marker gene expression and functionality.

##### Cryopreservation

1. Cryopreserved stem cells shall be clearly labelled with the cell line name, culture conditions, passage number, operator name and cryopreservation date. Cryopreserved cells shall have the same unique identification used during the process of harvesting, separation and expansion, etc.
2. The cryopreservation procedure shall follow the known principles of cryopreservation of mammalian cells.

##### Resuscitation

1. The resuscitation process shall be as rapid as possible to ensure the optimal viability and biological activity of stem cells.
2. Cell line information including but not limited to the cell line name, passage number, culture conditions, operator name, resuscitation date and time shall be documented and recorded.

### 6.3 Storage

1. Principles and standard operating procedures for the storage and management of stem cells shall be established and implemented. Detailed information including but not limited to all cell lines' documentation, application requests to the stem cell storage organization, and ethical reviews shall be documented and recorded.
2. Application requests of stem cell lines shall be submitted to and approved by the stem cell storage organization.
3. Stem cell lines stored in the stem cell bank shall be in accordance with the management requirements for stem cell banks.

[SOURCE: ISO 20387:2018, 3.5]

### 6.4 Transportation

1. The appropriate mode of transportation and transportation conditions shall be selected to ensure preservation of the optimal biological characteristics, safety, stability and efficacy of stem cells.
2. The transportation of stem cells shall consider the following factors, including but not limited to cell characteristics, the container, transportation routes, transportation conditions, transportation equipment, transportation methods, transportation risks and risk mitigation measures.
3. The management of transportation conditions shall include but not limited to temperature range, vibration, contamination, equipment performance and packaging.
4. The transportation documentation shall include but not limited to information on the mode and condition of transportation, the path, the duration time, the personnel, the shipping address and the stem cell line.

## 7 LABORATORY TESTING

1. Standard testing procedures and operational management rules shall be established according to specific purposes to ensure the accuracy and reliability of the testing process and testing results. The testing personnel shall fully understand and implement these documents.
2. The reagents, consumables and other supplies shall meet the requirements for stem cell testing.
3. The testing personnel shall be well‐trained for stem cell manipulation. Where there are special legal and regulatory requirements for the specific testing of stem cell, the testing personnel shall meet the corresponding requirements.
4. The facilities and environment for testing shall meet corresponding national regulations and standards, and ensure that all testing results are valid and of optimal quality.
5. The testing equipment shall meet the requirements for stem cell testing, and the equipment management procedures, including but not limited to the use and maintenance of equipment shall be established. The testing instrument shall be used within the validity period, and calibration shall be routinely performed for the key parameters that may influence the results to ensure that the testing equipment meets the testing requirements.
6. Management of testing reagents and hazards shall comply with the relevant regulations.
7. The biological characteristics, safety, stability, and efficacy of stem cells shall be tested and recorded during stem cell separation, expansion, differentiation, storage and resuscitation.

## 8 WASTE DISPOSAL

1. Management documents for disposal of stem cells shall be established and the management specifications shall be strictly implemented and recorded in detail.
2. Disqualified/discarded stem cells or their raw source materials (such as the donor's embryo, germ cells, bone marrow, blood, etc.) that arise during stem cell research and production shall be disposed according to legal and/or ethical regulations.

## CONFLICT OF INTEREST

No potential conflicts of interest are disclosed.

## AUTHOR CONTRIBUTIONS

QZ and TZ contributed to conception and design. JH, AM, LW, JC and SC drafted and revised the manuscript. LW, BF, JZ, XP, YZ, PX, SH, QL, YZ, YX, HZ, GS critically read and revised the manuscript.
